# Native Killer Yeasts as Biocontrol Agents of Postharvest Fungal Diseases in Lemons

**DOI:** 10.1371/journal.pone.0165590

**Published:** 2016-10-28

**Authors:** María Florencia Perez, Luciana Contreras, Nydia Mercedes Garnica, María Verónica Fernández-Zenoff, María Eugenia Farías, Milena Sepulveda, Jacqueline Ramallo, Julián Rafael Dib

**Affiliations:** 1 Planta Piloto de Procesos Industriales Microbiológicos - Consejo Nacional de Investigaciones Científicas y Técnicas, Tucumán, Argentina; 2 Instituto de Microbiología, Facultad de Bioquímica, Química y Farmacia, Universidad Nacional de Tucumán. Ayacucho 471 (4000), Tucumán, Argentina; 3 Laboratorio de Desarrollo e Investigación, SA San Miguel, Lavalle 4001, T4000BAB, San Miguel de Tucumán, Argentina; Tulane University Health Sciences Center, UNITED STATES

## Abstract

Economic losses caused by postharvest diseases represent one of the main problems of the citrus industry worldwide. The major diseases affecting citrus are the "green mold" and "blue mold", caused by *Penicillium digitatum* and *P*. *italicum*, respectively. To control them, synthetic fungicides are the most commonly used method. However, often the emergence of resistant strains occurs and their use is becoming more restricted because of toxic effects and environmental pollution they generate, combined with trade barriers to international markets. The aim of this work was to isolate indigenous killer yeasts with antagonistic activity against fungal postharvest diseases in lemons, and to determine their control efficiency in *in vitro* and *in vivo* assays. Among 437 yeast isolates, 8.5% show to have a killer phenotype. According to molecular identification, based on the 26S rDNA D1/D2 domain sequences analysis, strains were identified belonging to the genera *Saccharomyces*, *Wickerhamomyces*, *Kazachstania*, *Pichia*, *Candida* and *Clavispora*. Killers were challenged with pathogenic molds and strains that caused the maximum *in vitro* inhibition of *P*. *digitatum* were selected for *in vivo* assays. Two strains of *Pichia* and one strain of *Wickerhamomyces* depicted a significant protection (p <0.05) from decay by *P*. *digitatum* in assays using wounded lemons. Thus, the native killer yeasts studied in this work showed to be an effective alternative for the biocontrol of postharvest fungal infections of lemons and could be promising agents for the development of commercial products for the biological control industry.

## Introduction

Argentina is recognized worldwide for occupying one of the first places in the production, export and industrialization of lemons. Tucuman province is the most important production center, generating 73.26% of the total production. The main destination of lemon production in Argentina is the industry for the production of concentrated juice, essential oil and dehydrated peel, for internal and external consumption. However, approximately 21% is marketed as fresh fruit, which is mainly exported [1]. After lemons are harvested, they become vulnerable to attack by saprophytic pathogens or parasites, due to their high content of water and nutrients, and because they have lost most of the inherent strength that protects them during their development in the tree. Its organic acid content is sufficient to produce a pH lower than 4.6, which favours the development of fungi [2]. Thus, the economic losses caused by postharvest diseases currently represent one of the main problems of the citrus industry worldwide.

The main diseases that affect this group of fruits are the "green mold" and "blue mold", produced by *Penicillium digitatum* and *Penicillium italicum* respectively, being the first the most common and with a rather high reproductive activity [3]. The "blue mold" is less prevalent; however it becomes more important during cold storage because it thrives better than the "green mold" at low temperatures [4]. The main sources of contamination are conidia, which do not germinate until the skin of the fruit is damaged. Therefore, these fungi are wound pathogens and cause a soft and wet rot that quickly deteriorates the organoleptic characteristics of the fruit [5]. To control these diseases, the use of synthetic fungicides is the most commonly used method due mainly to its relatively low cost, ease of application and effectiveness; agents such as thiabendazole (TBZ) and imazalil (IMZ) are the most commonly used. Nevertheless, resistant strains occur at a high frequency, which limits their effectiveness [6]. Furthermore, their use is becoming more restricted due to their high residual toxicity, carcinogenic effects, degradation and generated environmental pollution [7,8]. Therefore, the increasing needs for methods of low environmental impact and minimal risk to human health, demands the development of novel alternative solutions. Among them, physical methods such as heat treatment or radiation or chemical methods using natural or synthetic chemicals with low residual and toxicological effects are known [9]. Furthermore, the controlled use of microorganisms which antagonize pathogenic microorganisms also shows great potential as an alternative method for controlling postharvest diseases [10]. Interestingly, most antagonistic microorganisms are isolated from the surface of the fruits, having adaptive advantages, and thus may be better antagonists than those from other sources [11].

Several mechanisms of action are involved in biological control processes. These mechanisms are generally based on the ability of biocontrol agents to: adhere to specific sites, including both yeasts and pathogenic cells [12]; colonize wounds and compete for nutrients; secrete specific enzymes [13]; induce resistance [14]; regulate the population density at specific sites [15]; secrete antimicrobial substances (soluble or volatile) and form a biofilm on the inner surface of wounds [16]. In particular, yeasts as biocontrol agents against postharvest pathogens, have certain advantages: i) they can grow rapidly in fermenters using inexpensive substrates and therefore easily producing large yields [17]; ii) non production of allergenic spores or mycotoxins, in contrast to filamentous fungi [18]; and iii) have simple nutritional requirements being able to colonize dry surfaces for long periods of time [19]. Special attention has been given to yeast displaying the “killer” phenotype, first described in *Saccharomyces cerevisiae* [20]. This killer activity can be defined as the ability of some yeast to secrete protein toxins or low molecular mass glycoproteins that are lethal to other susceptible yeasts [21] as well as to filamentous fungi and bacteria [22,23,24]. Various yeasts with such phenotype have shown high efficiency in the control of *Penicillium* and other pathogenic fungi that cause postharvest infections in fruits such as lemons, oranges and papaya [22,23,25]. However so far there was no work on isolation of indigenous killer yeasts for biological control in citrus of this region, which would have adaptive advantages for the vast production of lemons in Tucumán.

The aim of this work was to isolate and identify native yeasts with killer phenotypes from the province of Tucumán from the surface of leaves and fruits of different citrus, and wash-water from lemon shells from a local citrus industry, and evaluated their effectiveness against postharvest pathogens in lemons.

## Materials and Methods

### Samples

Fruits and leaves of citrus plants (lemon, orange, tangerine and grapefruit) were collected during September 2013 in the town of San Miguel de Tucumán, Argentina. The sampled trees had not received any pre-harvest treatment with synthetic products (pesticides, herbicides or artificial fertilizers). Moreover, samples from wash-water peel from a local citrus company in San Miguel de Tucumán were aseptically taken. A permission to conduct these studies was given by the local authority: “Dirección Flora, Fauna Silvestre y Suelos” (Department of Flora, Fauna and Soils), Tucumán, Resolution N° 67–16 (DFFSyS).

### Isolation of native yeast

Sterile wet cotton swabs were streaked on leaves and citrus fruit surfaces and immediately placed into 10 mL of YEPD (yeast extract 5 g/L, peptone 5 g/L, dextrose 40 g/L, pH 4.5–5.0, 0.1 buffered mol/L phosphate citrate buffer) supplemented with 100 mg/L ampicillin and 50 mg/L chloramphenicol to inhibit bacterial growth. Flasks were incubated at 28°C for 24 h with vigorous shaking (140 rpm). After incubation aliquots were plated on YEPD-agar supplemented with antibiotics. The isolation of yeasts from wash-water lemons were performed by direct seeding on YEPD-agar supplemented with ATB. All plates were incubated at 28°C for 48 h.

Yeast colonies were isolated from streaked YEPD plates according to their macroscopic features (texture, surface, margin, elevation and color) and yeast morphology was confirmed by microscopic observation.

### Determination of killer activity

Yeast killer activity of isolates was evaluated using two different protocols: eclipse technique [26] and diffusion plate technique described by Stumm et al. [27], with modifications. *Saccharomyces cerevisiae* CEN.PK2-1c [28] was used as a sensitive reference strain. *Kluyveromyces lactis* strain AWJ137 [29] and *S*. *cerevisiae* GS1731 [30] were used as positive and negative controls, respectively. Assays were performed on YEPD agar at a pH of 4.5, as this is the value closest to the optimum pH for the killer activity of most yeast species [31]. Since the action of the killer toxins produced by some yeast isolated from the environment depends on the presence of NaCl [32], the diffusion assay plate was performed with (2%) and without NaCl.

### Taxonomic identification of killer yeasts

#### DNA extraction

DNA extraction was performed according to the modified technique described by Fernandez Zenoff et al. [33]. Yeasts were grown in YEPD medium at 28°C under agitation (140 rpm) for 24 h and pelleted by centrifugation at 12,000 rpm. The supernatant was discarded and pellets were incubated for 1 h at 55°C with 0.75 mL of 2% [wt/vol] CTAB isolation buffer (Sigma) (1.4 M NaCl, 0.2% [vol/vol] 2-mercaptoethanol, 20 mM EDTA, 100 mM Tris-HCl, pH 8) and sterile glass beads (0.5 mm in diameter, Sigma). Every 15 min, samples were vigorously shaken. After extraction, samples were washed with 0.5 mL chloroform-isoamyl alcohol (24:1, vol/vol) and centrifuged (10 min, 12,000 rpm). DNA was precipitated with 0.5 mL of isopropanol (1 h, 4°C) followed by centrifugation (30 min, 12,000 rpm, 4°C). The pellets were washed twice with 80% cold ethanol, vacuum dried, and dissolved in 50 μL of TE buffer (10 mM Tris, 1 mM EDTA, pH 8.0). DNA samples were subjected to gel electrophoresis on 0.8% agarose (w/v) using TAE (Tris-acetate-EDTA) 1X as running buffer. Gels were stained with Syber Green dye (Invitrogen). Extracted DNA was stored at −20°C.

#### Molecular identification

Molecular identification was performed by PCR of the D1/D2 region from the rDNA 26S. PCR reactions were carried out using primers NL-1 (5’-GCA TAT CAA TAA GCG GAG GAA AAG-3’) and NL-4 (5’-GGT CCG TGT TTC AAG ACG G-3’) [34].

PCR amplification was performed as follows: 50–100 μg/μL of purified genomic DNA, 0.5 mM of each primer, 0.8 mM deoxyribonucleoside triphosphate (dNTP), 1.5 mM MgCl_2_, 2.5 μL of 10X (Taq polymerase) PCR buffer and 1.25 U of Taq DNA polymerase (Invitrogen) and sterile water to a final volume of 25 μL. The amplification conditions were as follows: initial denaturation at 94°C for 3 min, annealing temperature at 53°C for 45 sec, extension at 72°C for 90 sec, final extension at 72°C for 10 min. A total of 30 cycles were performed. Amplification products were analyzed by electrophoresis in 1% (wt/vol) agarose gels. Gels were stained with Syber Green according to the manufacturer's specifications. PCR products were sequenced in MAGROGEN (Korea). The obtained sequences were aligned using the BLAST analysis (http://www.ncbi.nlm.nih.gov/BLAST) for molecular identification.

### Determination of *in vitro* killer activity

The antagonistic effect of killer yeasts was evaluated against three common citrus phytopathogens: *P*. *digitatum*, *P*. *italicum* and *P*. *citri*, employing the technique described by de Lima et al. [22] with some modifications. Fungal strains used in this study belong to the fungi collection from the Plant Pathology Lab from “Estación Experimental Obispo Colombres” (EEAOC, Tucuman, Argentina).

Spore suspensions were prepared from a 10-day pure culture grown at 25°C on PDA medium (4 g/L potato extract, 20 g/L glucose, 15 g/L agar, pH 5.6). 3 mL of sterile distilled water was added on the surface of the mycelium and the suspension was made by scraping with a sterile loop. The suspension was transferred to a sterile test tube, which was vigorously stirred. Subsequently, the spore concentration was visually adjusted to 0.5 of the McFarland scale, which corresponds to approximately 1×10^8^ CFU/mL [35]. 5 μL of spore suspensions of each fungus was plated in the center of PDA plates. Then the yeast was plated at two adjacent sites, 3 cm far from to the central drop. The negative control consisted in PDA plates inoculated only with 5 μL of each fungus spores suspensions. Plates were incubated at 25°C for 10 days and diameters of each fungus growth were periodically measured. According to the diameters measurement data, the relative degree of growth inhibition against each phytopathogen was estimated.

Furthermore, the mycelia of phytopathogens that had been inhibited by the killer yeast was observed using a stereomicroscope (10X magnification), and compared with corresponding controls. A microscopic analysis of micelles was also performed. For that purpose, mycelial discs of about 5 mm in diameter, taken from the nearest area to yeast cells, were removed from the plates. Hyphae were photographed and their morphologies were compared against controls [23].

### *In vivo* antagonist activity against *P*. *digitatum*

The type of control that the killer yeasts performed on lemons was determined using a modification of the procedure described by Sepulveda et al. [36], using fruits without any postharvest treatment. Test involves a Primary Infection Control, a Secondary Infection Control and a Wound Protection Control (S1 Fig). Ten fruits were used in each treatment and the experiment was repeated 4 times. Data were subjected to the analysis of nonparametric variance (Kruskal Wallis) using InfoStat/E version 2015 [37] to assess statistical differences in growth inhibition of the pathogen within 5 days of treatment for different killer yeasts tested in relation to their respected controls. A 95% confidence level was used in the analysis.

#### Primary infection control (Curative Effect)

Fruits were disinfected with 70% ethanol and were wounded and inoculated in the equatorial zone with a suspension of 1×10^6^ spores/mL *P*. *digitatum* by using an awl. Wounds were about 2 mm deep and 1 mm wide. Lemons were incubated in a chamber at 24°C and high humidity (about 90%) for 24 h. In addition, 20 fruits were selected as controls, which were incubated under the same conditions.

5 L of each yeast suspension were prepared as detailed below, in which the inoculated fruits were dipped using net bags. After the fruits were drained and placed in honeycombed trays, they were covered with polyethylene bags and incubated for 5 days at the above described conditions. Controls were kept under the same conditions but without being treated with yeasts.

#### Secondary infection Control (Preventive Effect)

In this case, the treatment with yeast was first done on the fruits and then a *P*. *digitatum* inoculation was performed as seen in S1 Fig. Twenty control fruits, which were not subjected to any yeast treatment, were also inoculated with the pathogen.

#### Wound protection control

The disinfected fruits were wounded with an awl and then immersed in the yeast suspension as described above. After 24 h of incubation at 24°C and high humidity, the wounded and treated fruits were immersed in 5 L of a spore suspension of *P*. *digitatum* (1x10^6^ spores/mL) for 1 min using net bags and incubated for 5 days at the previously described conditions. 20 fruits were used as a control; they were wounded and only submerged in the suspension of the pathogen.

#### Protection of wounds at low temperature

The ability of killer yeast strains to control the disease caused by *P*. *digitatum* at low temperature was tested as well. The assay described for wound protection test was conducted but modifying the temperature and time of incubation. The wounded fruits treated with the yeast were also incubated at 24°C and high humidity for 24 h. After being immersed in the suspension of spores, they were stored in a chamber at 7°C, and monitored at 5, 14 and 21 days. Four repetitions of 15 fruits were performed.

#### Yeast and fungal spore suspension preparation

A yeast preinoculum was prepared in flasks with 25 mL of YEPD medium and incubated at 28°C at 160 rpm for 24 h. Six Erlenmeyers containing 250 mL YEPD medium were inoculated with 2.5 mL of preinoculum, and then incubated at 28°C at 160 rpm for 48 h. Cells were collected by centrifugation at 10,000 rpm for 10 min and washed twice with sterile saline solution. For each yeast, 15 L of cell suspension was prepared in distilled water after growth in 1.5 L of YEPD medium.

A spore suspension (1x10^6^ spores/mL) was prepared from fruit infected with *P*. *digitatum* and the concentration was determined by counting the cells using a Neubauer chamber.

### *In vivo* antagonistic activity against *P*. *italicum*

To determinate *in vivo* antagonist activity against *P*. *italicum*, the technique described by Platania et al. [23], which is basically a wound protection control, was used. Yeast suspensions to be tested were prepared from overnight cultures (28°C, 160 rpm). 1 mL of each culture was centrifuged for 5 min at 12,000 rpm and the supernatant was discarded. The pellet was washed twice with sterile water and then resuspended in 1 mL. Lemons were washed and disinfected with 70% ethyl alcohol. Three wounds of about 2 mm deep and 1 mm wide were made along the mid-line with a previously disinfected awl. Four lemons were randomly selected for treatment with each yeast strain and four were selected to be used as a control. Lemon wounds were then inoculated with 10 μL of yeast suspension and were allowed to air dry. No inoculation was made in control fruits. All lemons were placed in plastic trays wrapped with plastic bags containing water-soaked cotton to maintain moisture, and stored at 24°C for one day. Afterwards, the lemon wounds were inoculated, including the control fruits, with 10 μL of a *P*. *italicum* spore suspension (1×10^8^ spores/mL) and allowed to air dry. Lemons were placed under the same conditions as described above and stored at 20°C. They were observed for the presence of disease after 5 and 7 days. All assays were done in triplicate. A non-killer control yeast (*S*. *cerevisiae* CEN.PK2-1c) was additionally tested following the same procedure as described before.

### Inhibitory activity against human pathogenic bacteria

Inhibitory activity of the selected killer yeast was determined against pathogenic bacteria using the procedures of deferred antagonism as described by Gratia [38] and Fredericq [39]. Reference strains (S3 Table) were kindly provided by the Laboratory of Bacteriology from “Instituto de Maternidad y Ginecología Nuestra Señora de las Mercedes” (San Miguel de Tucuman city). 5 μL of each yeast active cultures were plated in a thin layer of medium YEPD and incubated at 25°C for 2 days. Bacteria strains were grown in LB medium (10 g/L tryptone, 5g/L yeast extract, 10 g/L NaCl; pH 7) at 37°C with shaking (180 rpm) to reach 10^7^ CFU/mL. Then, each culture was mixed with 15 mL of melted Muller-Hinton agar (300 g/L meat infusion; 17.5 g/L peptone acid casein; 1.5 g/L starch; 15 g/L agar) and it was placed above the layer containing grown yeasts. Halos formed around the yeasts after 24 h at 37°C indicated the presence of antimicrobial compounds.

### Plasmid extraction

Plasmid extraction was performed using the technique described by Kaiser et al. [40]. 1 mL of each overnight yeast culture (28°C, 160 rpm) was collected by centrifugation, washed with 1 mL of sterile distilled water and resuspended in 140 mL of solution 1 (35 mM Tris/HCl, 1.2 M sorbitol, 100 mM EDTA, 46 μL/100 mL β-mercaptoethanol; pH 7.5). After 30 min incubation at 30°C, cells were pelleted by centrifugation and resuspended in 15 μL of solution 2 (50 mM Tris/HCl; 30 mM EDTA; pH 7.5). 1 μL of Zymolase (5 U/μL) was added and incubated at 30°C for 1 h to remove the cell wall chitin. Then, 2 mL of 20% (w/v) SDS solution and 3 μL of Proteinase K (20 mg/ml) was added to lyse the protoplasts and to remove terminal proteins. Lysis and Proteinase K digestion were performed at 50°C for 3 h. Cell debris were removed by centrifugation (13000 rpm, 10 min). After performing a 1% agarose gel electrophoresis at 80 V for 2 h (1X TAE), plasmids were visualized using a transilluminator. *Kluyveromyces lactis* AWJ137 was used as a positive control, which contains two linear plasmids [41].

## Results

### Isolation

A total of 437 native yeast strains were isolated from leaves and fruits of citrus plants (free from fungicide or other substances of synthetic origin) and wash-water from lemon shells from a local citrus company. S1 Table shows the number of strains isolated from different sources. Most yeasts (160) were isolated from the lemons wash-water.

### Killer activity

All isolates underwent two different tests to determine the killer phenotype. Using the eclipse assay, 22 yeast strains (5%) showed a killer phenotype against sensitive reference strain *S*. *cerevisiae* CEN.PK2-1c. When the diffusion technique in plates was applied, 30 yeast strains (6.9%) displayed a killer phenotype. Finally, when the diffusion plate was carried out with the addition of 2% NaCl, 37 yeast strains (8.5%) showed to be of the killer phenotype. S2 Table shows yeast strains with killer activity according to the different techniques used and the different isolation sources. The diffusion technique allowed for the identification of eight more killer strains than those determined with the eclipse assay from strains isolated from wash-water shells lemons. Seven additional strains with killer activity were obtained when the plate diffusion technique was made with 2% NaCl compared to the assay without NaCl. As it is shown in S2 Fig, the growth inhibition halos produced by killer strains showed larger diameters with the addition of 2% NaCl, regarding the halos produced by the same strains in the absence of NaCl. None of the isolates from lemons, oranges and grapefruit plants showed killer activity using any of the tested techniques.

### Identification

Phylogenetic analysis of yeast strains showing killer activity was performed by analyzing the D1/D2 sequence of 26S rDNA gene. Six different genera were identified: *Pichia* (8.1%), *Saccharomyces* (13.5%), *Kazachstania* (40.5%), *Wickerhamomyces* (2.7%), *Clavispora* (8.1%) and *Candida* (21, 7%). Table 1 shows the identified killer yeasts.

**Table 1:** Yeast identification according to 26S rDNA gene D1/D2 sequence.

Strain	Identification	Similarity (%)
**5**	NI *	
**27**	*Pichia fermentans*	100
**28**	*Pichia fermentans*	100
**41**	*Pichia fermentans*	99
**42**	*Saccharomyces cerevisiae*	100
**50**	*Kazachstania exigua*	99
**56**	*Wickerhamomyces anomalus*	100
**65**	*Kazachstania exigua*	99
**68**	*Candida catenulata*	100
**73**	*Kazachstania exigua*	100
**84**	*Kazachstania exigua*	99
**88**	*Kazachstania exigua*	99
**93**	*Kazachstania exigua*	99
**94**	*Kazachstania exigua*	99
**95**	*Kazachstania exigua*	99
**97**	*Kazachstania exigua*	99
**113**	*Kazachstania exigua*	99
**116**	*Saccharomyces cerevisiae*	100
**120**	*Kazachstania exigua*	99
**122**	*Kazachstania exigua*	99
**123**	*Candida pararugosa*	100
**124**	*Kazachstania exigua*	99
**125**	*Saccharomyces cerevisiae*	100
**131**	*Kazachstania exigua*	99
**132**	*Kazachstania exigua*	99
**137**	*Saccharomyces cerevisiae*	99
**145**	*Saccharomyces cerevisiae*	100
**146**	*Clavispora lusitaniae*	100
**147**	*Clavispora lusitaniae*	100
**160**	*Clavispora lusitaniae*	100
**M1.1**	*Candida catenulata*	100
**M1.2**	*Candida catenulata*	100
**M1.3**	*Candida catenulata*	100
**M1.4**	*Candida catenulata*	100
**M1.5**	*Candida catenulata*	100
**M1.6**	*Candida catenulata*	100
**M1.7**	NI	

* NI: not identified.

### *In vitro* antagonism assays

**Fig 1:**
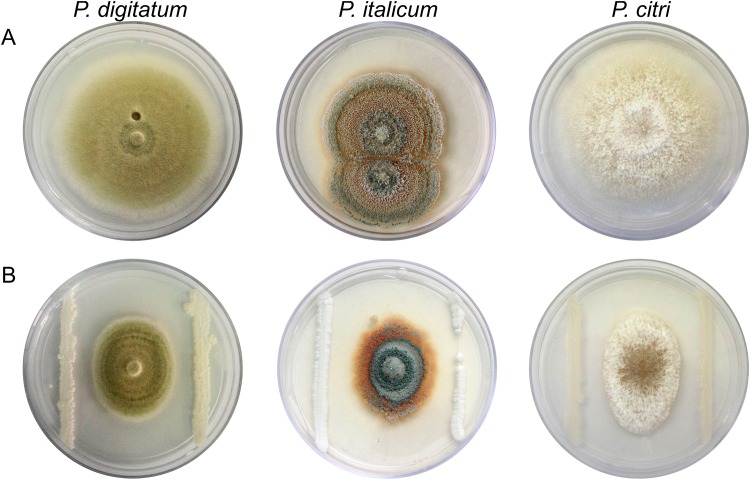
*In vitro* inhibitory activity of killer strain 27 against *P*. *digitatum*, *P*. *italicum* and *P*. *citri* on PDA medium after 10 days incubation at 25°C. (A) Control plates inoculated only with plant pathogens. (B) Plates inoculated with killer strain 27 and plant pathogens.

Antagonism activity of killer yeasts was studied against *P*. *digitatum*, *P*. *italicum* and *P*. *citri* on PDA medium. Activity was determined by calculating the percent of relative growth inhibition of each fungus; the growth diameters of the three phytopathogens exposed to 37 killer yeasts and their respective controls were used as reference data, after 10 days of incubation (Fig 1). The results obtained are shown in Table 2. In the case of *P*. *digitatum*, 13 yeast strains caused growth inhibition equal to or greater than 40%; 14 strains caused an inhibition between 16 and 39%; in the remaining 10 strains, inhibition was equal or less than 15%. In relation to *P*. *italicum*, 11 strains caused growth inhibition equal to or greater than 40%; 18 strains caused an inhibition between 16 and 39%; and the remaining 8 strains were less than or equal to 15% inhibition. For *P*. *citri*, 19 strains caused growth inhibition equal to or greater than 40%; 11 strains caused an inhibition between 16 and 39%; and the remaining seven strains inhibition was less than or equal to 15% growth.

**Table 2:** Degree of relative inhibition of killer yeast strains against *P*. *digitatum*, *P*. *italicum* and *P*. *citri*.

Killer yeast strains	*P*. *digitatum*	*P*. *italicum*	*P*. *citri*
**5**	+++	++	++
**27**^a^^,^^b^	++	+++	++
**28**^a^^,^^b^	+++	+++	++
**41**	++	+	+++
**42**	++	+	+
**50**	++	++	+++
**56**^a^^,^^b^	++	+++	++
**65**	+	+++	++
**68**	+	++	++
**73**	+	++	+++
**84**	+	++	+++
**88**	+++	+	+++
**93**	+++	++	+++
**94**	++	+++	+++
**95**	+++	++	+++
**97**	+	++	+
**113**	+++	++	+++
**116**	++	++	++
**120**^b^	++	++	+++
**122**	+++	++	+++
**123**	++	+	+
**124**	++	+++	+++
**125**	+++	++	++
**131**	++	+++	+++
**132**	+++	++	+++
**137**^b^	+++	+++	+++
**145**	++	+	+
**146**	+++	++	++
**147**	+++	++	+
**160**	+++	++	+
**M1.1**	+	+++	+
**M1.2**	+	+	++
**M1.3**	+	+	+++
**M1.4**	++	+++	+++
**M1.5**	+	+++	+++
**M1.6**	++	+	+++
**M1.7**	+	++	++

^+++^: Growth inhibition ≥ 40%;

^++^ Growth inhibition between 16 and 39%;

^+^: Growth inhibition ≤ 15%.

^a^, selected for *in vivo* protection tests against *P*. *digitatum*.

^b^, selected for *in vivo* protection tests against *P*. *italicum*.

According to these tests, the yeast 137 was the only yeast that caused an inhibition equal to or greater than 40% in the three plant pathogens; yeast 28 caused an inhibition greater than or equal to 40% of *P*. *digitatum* and *P*. *italicum*; 88, 93, 95, 113, 122 and 132 strains showed an inhibition equal to or greater than 40% against *P*. *digitatum* and *P*. *citri*; 94, 124, 131, M1.4 and M1.5 strains showed an inhibition equal to or greater than 40% of *P*. *italicum* and *P*. *citri*.

**Fig 2:**
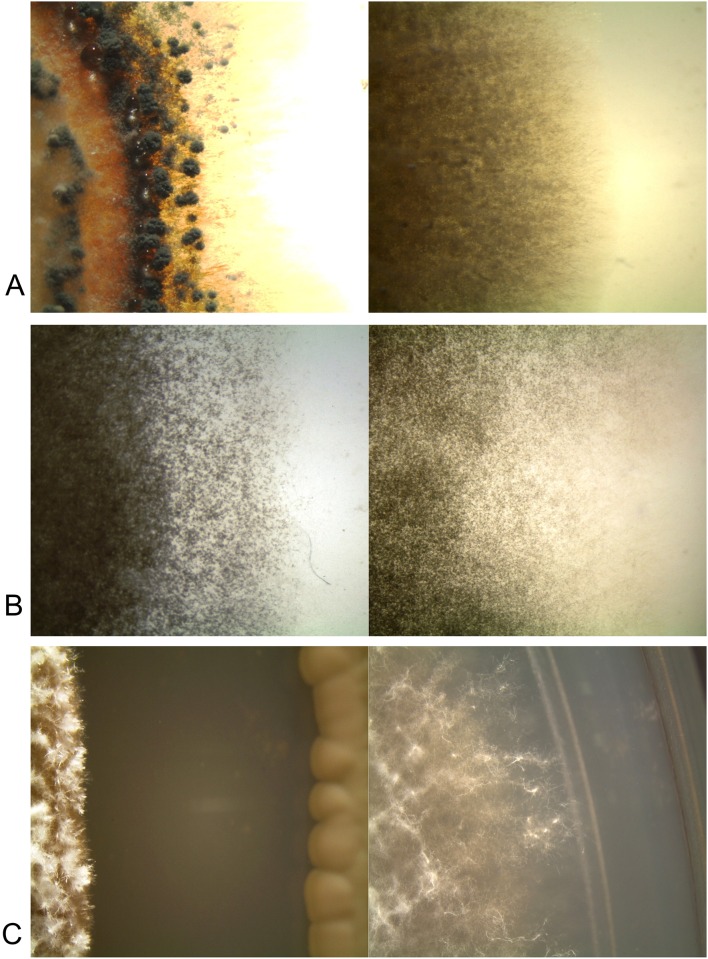
*In vitro* inhibition of killer yeast 137 against fungi on PDA medium (10 days of incubation at 25°C) under loupe (10X magnification). To the left is shown the mycelium from *P*. *italicum* (A), *P*. *digitatum* (B), and *P*. *citri* (C) inhibited by killer yeast 137. To the right control mycelia from each fungus are shown.

On the tenth day of incubation an observation through a stereomicroscope of aerial mycelia that experienced the greatest growth inhibition (≥ 40%) was conducted. They were compared with the corresponding controls to detect macroscopic differences. In general, *P*. *digitatum* and *P*. *italicum* mycelia inhibited by killer yeast strains were more compact, and in the case of *P*. *italicum*, spore clusters were observed; control hyphae were mostly extended towards the edges of the plate and spores were dispersed (Fig 2A and 2B). The aerial mycelium of *P*. *citri* showed a lower height and was less dense than the control mycelia, its hyphae were shorter and compact in the area closer to killer yeast (Fig 2C). Furthermore, any clear morphological differences in the hyphae of the fungus inhibited by killer yeast were found under microscopic analysis.

### *In vivo* antagonism tests against *P*. *digitatum*

#### Types of control of killer yeasts

**Fig 3:**
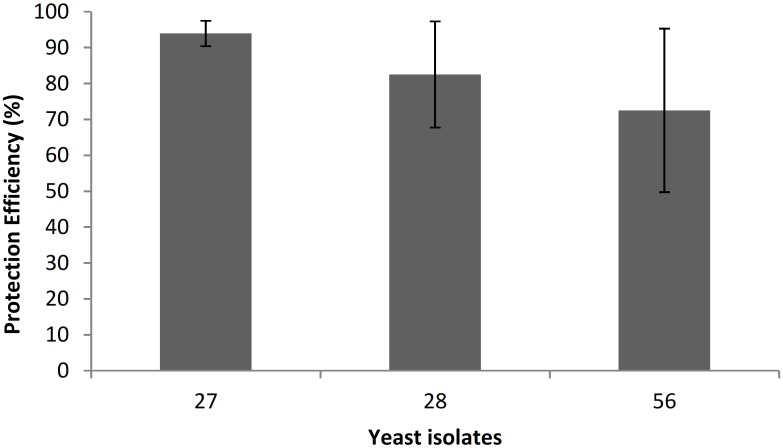
Efficiency of 27, 28 and 56 killer yeast strains in the wounds protection control against *P*. *digitatum*. Error bars indicate standard deviations.

**Fig 4:**
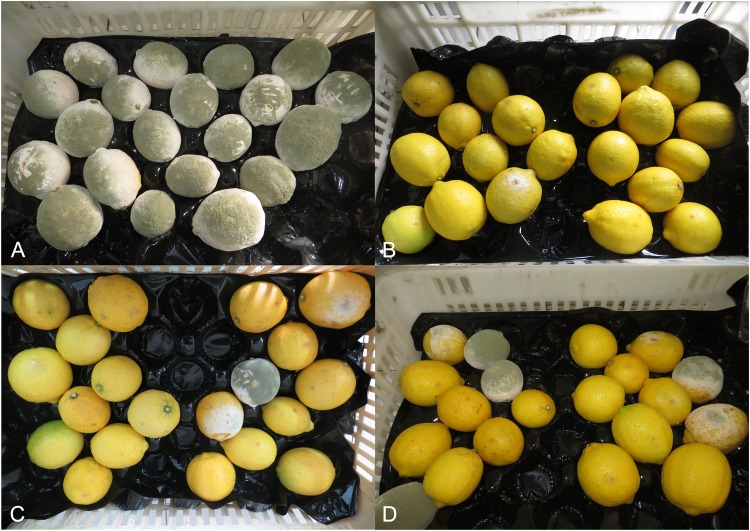
Control of *P*. *digitatum* by wounds protection with killer yeasts, after 5 days of incubation. (A) Control lemons, inoculated with pathogen spore suspension. (B), (C) and (D) pretreated lemons with 27, 28 and 56 yeast strains, respectively.

Based on *in vitro* antagonist activity results, 27, 28 and 56 killer yeast strains were selected to evaluate the types of control that performed against *P*. *digitatum*. None of the tested yeast strains succeed to control primary and secondary infections. However, in the wound protection control, after the fifth day of inoculation, the strains caused significant growth inhibition (p <0.05) of *P*. *digitatum*. Efficiencies of 93.6%, 82.5% and 72.5% were determinated for 27, 28 and 56 strains, respectively (Figs 3 and 4).

#### Wound protection at low temperature

**Fig 5:**
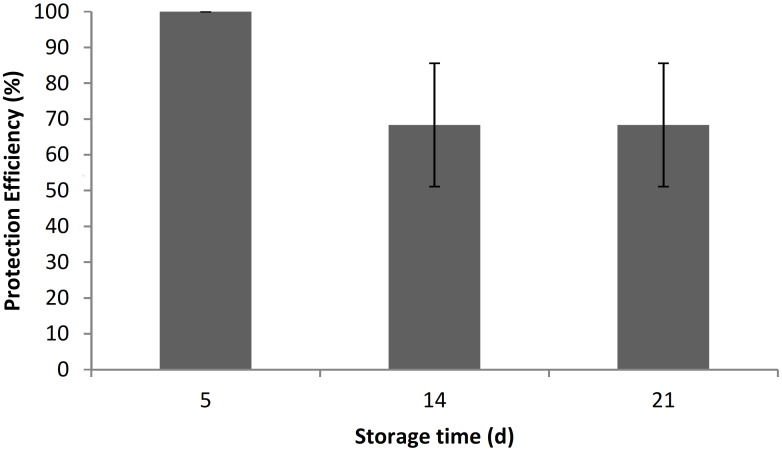
Wound protection control at low temperature of 27 strain against *P*. *digitatum*. Error bars indicate standard deviations.

To simulate the lemon shipping conditions during exportation to European and Asian markets, wound protection tests at 7°C was performed using strain 27, which showed to be the best candidate for protecting wounds at 24°C. In this case, during the first 5 days of storage at 7°C, no development of infection on yeast-pretreated fruits was observed. After 14 and 21 days, the efficiency of control over the plant pathogen was 68.33%. Although this does not represent a significant growth inhibition (p >0.05) of infection by *P*. *digitatum*, it was clearly observed that at the end of the assay there was no progression of the infection and the percentage of healthy lemons remained throughout the time course (Fig 5).

### *In vivo* antagonism tests against *P*. *italicum*

**Fig 6:**
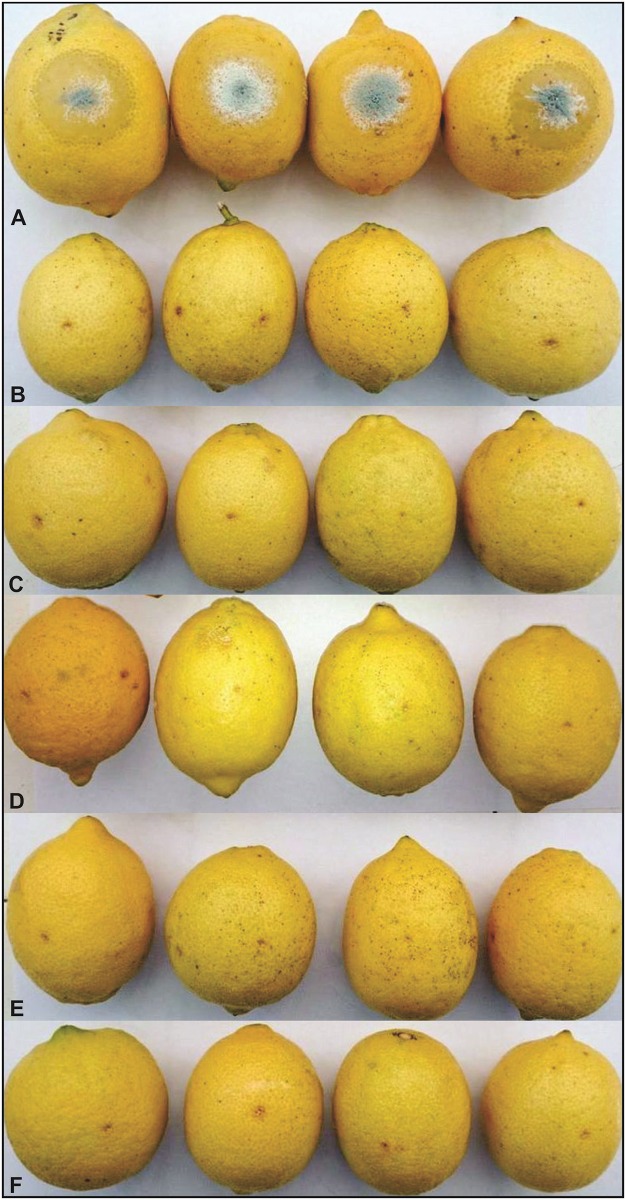
*In vivo* antagonistic effect of killer yeast against P. *italicum* observed after seven days of incubation. (A) Control fruit, only inoculated with the plant pathogen. (B), (C), (D), (E) and (F) correspond to pretreated lemons with 27, 28, 56, 120 and 137 yeast strains, respectively.

Protective activity of five yeast strains belonging to *Pichia* (27 and 28 strains), *Wickerhamomyces* (56), *Kazachstania* (120) and *Saccharomyces* (137), against *P*. *italicum* were determined. Since *P*. *italicum* showed a slower growth rate as compared to *P*. *digitatum* during the first 5 days, no apparent signs of decay in fruit treated with the killer yeast or in control lemons were observed. After 7 days, it was observed that all tested killer strains protected lemons against *P*. *italicum* infection (Fig 6). Meanwhile, in the same *in vivo* assay performed with *S*. *cerevisiae* CEN.PK2-1c (non killer phenotype yeast), no protection against the phytopathogen was observed (S3 Fig).

### Inhibitory activity against human pathogenic bacteria

Inhibitory activity of five killer strains was determined against human pathogenic bacteria using a procedure of deferred antagonism. Only strain 120, belonging to the *Kazachstania* genus inhibited the development of three of the five tested pathogens: *P*. *aeruginosa*, *E*. *coli* and *K*. *pneumoniae*. However, other tested yeasts showed no inhibitory effects (S3 Table).

### Plasmids extraction

Since killer phenotype in yeasts can be encoded in plasmids, isolation for these elements in killer yeasts was performed. Plasmids were observed in 42, 125, 137 and 145 strains. As it shown in S4 Fig, the size of these elements was circa 4 kb.

## Discussion

Given the global significance of lemon production in Argentina, particularly in the province of Tucuman, it is of great importance to find strategies to reduce costs and economic losses due to contamination by fungi and also give added value to the production. As it was mentioned above, the use of synthetic fungicides in the postharvest treatment of lemons shows drawbacks like the appearance of resistant strains. Likewise, because of their toxic and carcinogenic characteristics they constitute a hazard to both the environment and the health of humans. That is why, in addition to market restrictions in the amount of permitted chemical residues in fruit and the growing demand for organic products by consumers, the search for less hazardous alternatives takes strength and, at this point, the use of antagonistic microorganisms, such as killer yeasts, is distinguished by their offered advantages.

Lemon peel has many traditional uses, particularly in medicine and for its antioxidant properties [42]. Besides that, the essential oil represents a significant economic increase in citrus industry products [43]. However, the main disadvantage is the accumulation of fungicide residues which mainly occurs at skin level, where the removal is quite difficult by simple washing. Therefore the development of non-toxic safer alternatives for controlling postharvest diseases becomes more important.

Typically, a potential limitation on the use of biocontrol agents could be found: the adaptability to the conditions prevailing in each fruit and in environments where they are stored. In this sense, the selection and use of native antagonistic microorganisms isolated from the same ecological niches, became an effective strategy to prevent postharvest diseases caused by pathogenic fungi [22].

From the collection of yeast strains initially isolated, 8.5% were classified as killer yeast using different protocols. All techniques were performed at pH 4.5, being the closest to the optimum pH for most killer toxins [31]. Woods and Beban [44] first observed a dependence for killer activity associated with pH. Since then, the optimum pH has been defined for the activity of several killer toxins, with the majority of them having an optimum value between 4.2 and 4.7 [45,46]. Furthermore, the fact that most killer toxins are active at acidic pH levels, this may represent an advantage over traditional fungicides in biocontrol. In citrus fruits, pH in wounds decreases (lemon natural pH to 2 mm depth is about 5). This pH change dramatically alters the effectiveness of chemical fungicides as neutral forms penetrate the membrane of pathogens and are more toxic than ionized forms [47]. Siegel et al. [48] showed that imazalil (IMZ) was more toxic to *P*. *italicum* at pH 7 than pH 5.

The plate diffusion technique proved to be more sensitive than the eclipse assay for the determination of the killer activity. Moreover, the addition of NaCl enhanced sensitivity and an increase in the diameters of the inhibition halos was as well observed. This agrees with previous studies where it was observed that the activity of killer toxins produced by certain yeast strains depends on NaCl concentration. Lopes and Sangorrín [49] observed an increase in the diameter of the zones of inhibition caused by killer strains of *Wickerhamomyces anomalus*, *Metschnikowia pulcherima* and *Torulaspora delbrueckii* in the presence of 1 and 3% NaCl (w/v) with respect to control (without NaCl), and even in some cases killer activity was evidenced only when NaCl was added. The increase in killer activity in the presence of NaCl is the result of increased sensitivity of the target cells. Although the reasons behind such an increase in sensitivity is not clearly understood, it is known that certain killer toxins induce the formation of ion channels permeable through the plasma membrane. It has been suggested that the disruption of ionic equilibrium across plasma membrane in cells represents functional damage that can lead to an increase in mortality of poisoned cells in the presence of NaCl [50].

In this work we have observed that most of the *Kazachstania exigua* strains effectively inhibited the *in vitro* development of the three phytopathogens tested, especially *P*. *citri*. It is the first time a killer phenotype is described in this species (formerly *Saccharomyces exiguous*, [51]), which is considered as GRAS (Generally Recognized As Safe) microorganism [52]. Thus far, this species has been poorly characterized from a technological standpoint, however, as a biologically safe microorganism, it could be used as biological control agent in the food industry. Regarding other yeast species described in this work, they have already proved to be biologically safe, like for the species of *Saccharomyces*, *Pichia* and *Wickerhamomyces* [23, 53, 54]. Anyway, further studies should be performed concerning the safety of species like *C*. *catenulata*, *C*. *pararugosa* and *C*. *lusitaniae* for which infections in immunocompromised patients have been described [55, 56, 57].

Strain 56 was the only killer strain belonging to the genus *Wickerhamomyces* and was able to cause maximum *in vitro* growth inhibition of *P*. *italicum*. Additionally, it also inhibited (16–39%) the growth of *P*. *digitatum* and *P*. *citri*. There are precedents on the potential effect exerted by some killer fungal strains belonging to the genus *Wickerhamomyces* against postharvest pathogenic fungi in citrus. In a previous study by Platania et al. [23], killer strains of *W*. *anomalus* were capable of significantly inhibiting *in vitro* growth of *P*. *digitatum*. Izgu et al. [25] also observed *in vitro* inhibition of growth of *P*. *digitatum* and *P*. *italicum* by action of the killer toxin Panomycocin from *Pichia anomala* NCYC 434 (reclassified as *W*. *anomalus*). In addition, certain killer strains of *W*. *anomalus* significantly inhibited the growth of *Colletotrichum gloesporoides* on solid medium, the causal agent of anthracnose [22].

Furthermore, we identified three strains belonging to the genus *Pichia* (27, 28 and 41) which successfully inhibited the development of phytopathogenic fungi. To date, the antifungal activity of killer yeast of the genus *Pichia* in citrus fruits has not been reported. However, the antagonistic effect of killer strains of this genus was evaluated against postharvest pathogenic fungi from other fruits [53].

Among the killer strains belonging to *S*. *cerevisiae*, strain 137 was the most effective. It caused the highest *in vitro* inhibition of the three phytopathogenic fungi, whereas strain 125 of the same species, inhibited only *P*. *digitatum*. Platania et al. [23] tested the antagonistic effect of *S*. *cerevisiae* strains against *P*. *digitatum in vitro* and only observed a slight growth inhibition. Nowadays, the killer phenomenon of this species has become significant in the wine industry with an increasing interest to employ killer yeasts of *S*. *cerevisiae* as starter cultures, which would ensure the production of quality controlled wines and preventing the development of contamination by other yeasts [54,58].

Three killer yeast strains of the genera *Pichia* (27 and 28) and *Wickerhamomyces* (56) were chosen for *in vivo* assays to determine which of these yeasts would be best suited in controlling the growth of postharvest pathogenic microorganisms on citrus.

According to the fruit control assays, yeasts 27 and 28 (*Pichia*) and 56 (*Wickerhamomyces*) protect the wounds of the fruits, preventing colonization by pathogenic fungi. Therefore, these results suggest killer yeast 27, 28 and 56 represent potential biocontrol agents against the major postharvest diseases in lemons, being strain 27 the best candidate as it reached a control efficiency of 93,6%. Similar yeasts have also been widely described by other authors as biocontrol agents of plant pathogens [59,60,61]. Santos and Marquina [53] described the effects of a killer strain of *Pichia membranifaciens* in the biocontrol of *Botrytis cinerea* in grapevines (*Vitis vinifera*). In *in vivo* assays, by inoculating the plants with a mixture of spores of the pathogen and yeast cells, they found a 100% of viable specimens was obtained while using the purified killer toxin, 80% of the plants did not develop the disease. In addition, the exo-β-1,3-glucanase Panomycocin produced by *P*. *anomala* strain NCYC 434 showed a full protection on fresh lemons against *P*. *digitatum* and *P*. *italicum* even after 5–7 days of incubation [25]. Moreover, Platania et al. [23] reported the biological control of *P*. *digitatum* in Tarocco oranges using killer yeast strains of *Saccharomyces cerevisiae* and *Wickerhamomyces anomalus*.

On the other hand, we have found strains of *S*. *cerevisiae* (137) and of *K*. *exigua* (120) which work in lemon protection against attack by *P*. *italicum*. As already mentioned, there are no reports about such species with antagonistic activity against *P*. *italicum*.

Most screening studies of antagonistic microorganisms as control agents of postharvest diseases in fruits have been carried out at room temperature, being those conditions in which usually fruits are stored until marketing and subsequent consumption. The ability of the strain 27 (*Pichia*) to protect lemons from *P*. *digitatum* at low temperature (7°C) was studied, in order to simulate conditions in which they are subjected during export phase to countries in Europe and Asia. While the efficiency of protection from this killer yeast was lower with respect to the assay at 24°C, between the second and third week the disease progression completely stopped, which means no variation on the ratio of the number of sick vs. healthy lemons. Similar studies reported an incidence in reduction of phytopathogenic *Penicillium expansum* up to 33% on pears and the diameter of the lesions by 88%, by strains of *Aureobasidium pullulans* and *Rhodotorula mucilaginosa* after 60 days incubation at—1/0°C and 95% humidity [62]. Another yeast strain belonging to the species *Leucosporidium scottii*, isolated from Antarctic soils, was identified as a good biocontrol agent against postharvest pathogens of apples (*P*. *expansum* and *B*. *cinerea*) stored at low temperatures [63]. Moreover, it was found that *Leucosporidium scottii* produces soluble antifungal substances that inhibit *P*. *expansum* but not *B*. *cinerea*, whereas on the other hand, the volatile compounds have shown antifungal activity against both.

When the killer phenomenon in yeasts was first discovered, it was thought to be effective only against other yeasts, however a broader spectrum towards filamentous fungi and bacteria has been depicted [64,65]. We have shown strain 120 (*Kazachstania exigua*) to have inhibitory effects against pathogenic bacteria *P*. *aeruginosa*, *E*. *coli* and *K*. *pneumoniae*. This is the first report concerning the inhibitory activity against bacteria for this yeast. Currently, we are studying in detail the inhibitory compound generated by this strain, its nature as well as its mechanism of action.

Killer toxins can be encoded on linear plasmids like for *Kluyveromyces lactis* [41], which served as a positive control in plasmid extraction performed in this work. Gunge and Sakaguchi [41] demonstrated the above by gene transfer assays of plasmids from *K*. *lactis* into a toxin sensitive non-killer yeast strain, *S*. *cerevisiae*. Subsequently, the recipient strain expressed the same killer phenotype as that of the donor strain and also became resistant to the toxin itself. After curing of the plasmids, the recipient strain lost its killer activity and resistance to external killer toxin. From these results it was concluded that the killer and resistance genes were located on the same plasmid. Also, in *Wingea robertsiae*, linear plasmids are associated with a killer phenotype (pWR1A of 8.3 kb and pWR1B of 14.6 kb) [66]. Therefore, as preliminary studies have indicated that linear plasmids are related to killer phenotypes observed in yeasts isolated in previous works, in this study, we sought to determine the presence of such plasmids, having found extrachromosomal elements in four strains belonging to the species *Saccharomyces cerevisiae* (42, 125, 137 and 145). Furthermore, the absence of these genetic elements in other tested yeasts suggests that the killer phenotype could be encoded at chromosomal level.

Native killer yeasts proved to be effective agents for biocontrol of postharvest diseases of lemons and may be used as a safer alternative to the application of synthetic fungicides. We are currently studying the molecular mechanisms that are associated with the bioprotection effects of yeasts on lemons. Furthermore, the protective effects in other citrus or fruits that have reported the attack by phytopathogens belonging to the genus *Penicillium* will also be evaluated.

## Supporting Information

S1 Fig Fruit control assays. This test was performed to determine type of control of killer yeasts in lemons against *P*. *digitatum*.
(PNG)

S2 Fig Plate diffusion assay in YPD medium (pH 4.5) to determine killer activity without (A) or with 2% NaCl (B). Each spot corresponds to a yeast, which is tested at the same location in both petri dishes. *S*. *cerevisiae* strain CEN.PK2-1c was used for the lawn on plates. Numbers correspond to killer strains listed on Table 1. Positive Control (C +): *K*. *lactis* AWJ137; Negative control (C-): *S*. *cerevisiae* GS1731.
(JPG)

S3 Fig *In vivo* test with no killer yeast phenotype against *P*. *italicum*. Results after 7 days of incubation. (A) Pretreated lemons with *S*. *cerevisiae* CEN.PK2-1c (no killer yeast phenotype) and then inoculated with the plant pathogen. (B) Control lemons inoculated only with *P*. *italicum*.
(JPG)

S4 Fig Plasmid extraction on 1% agarose gel electrophoresis (80 V—2 h). M: molecular marker (1 kb DNA Ladder, Genbiotech). (A) Lane 1, yeast 5; 2, 27; 3, 28; 4, 41; 5, 42; 6, 56; 7, 120; 8, 122; 9, 124; 10, 125; 11, 132; 12, 146. (B) Lane 1, yeast 50; 2, 73; 3, 95; 4, 123; 5, 137; 6, 145.
(JPG)

S1 Table Isolation source and number of strains isolated. (DOCX)

S2 Table Yeast strains with killer phenotype according to the isolation source and employed method for killer activity. (DOCX)

S3 Table Inhibitory activity of killer yeasts against human pathogenic bacteria. (DOCX)
