# Supplementary figures and images for: Native Killer Yeasts as Biocontrol Agents of Postharvest Fungal Diseases in Lemons
Source: PLoS One. 2016 Oct 28;11(10):e0165590. doi: 10.1371/journal.pone.0165590 (PMC5085023; doi:10.1371/journal.pone.0165590)

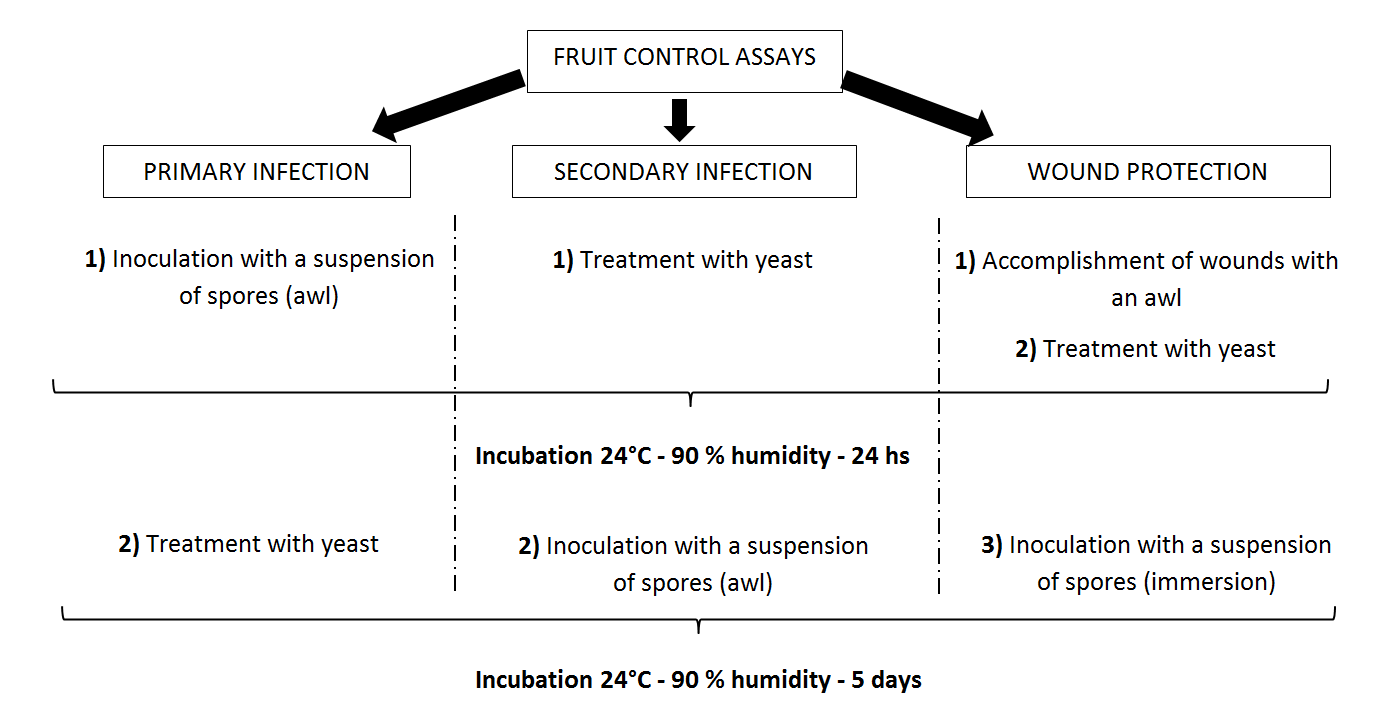

Supplement: S1 Fig — This test was performed to determine type of control of killer yeasts in lemons against P. digitatum. (PNG) [file pone.0165590.s001.png]

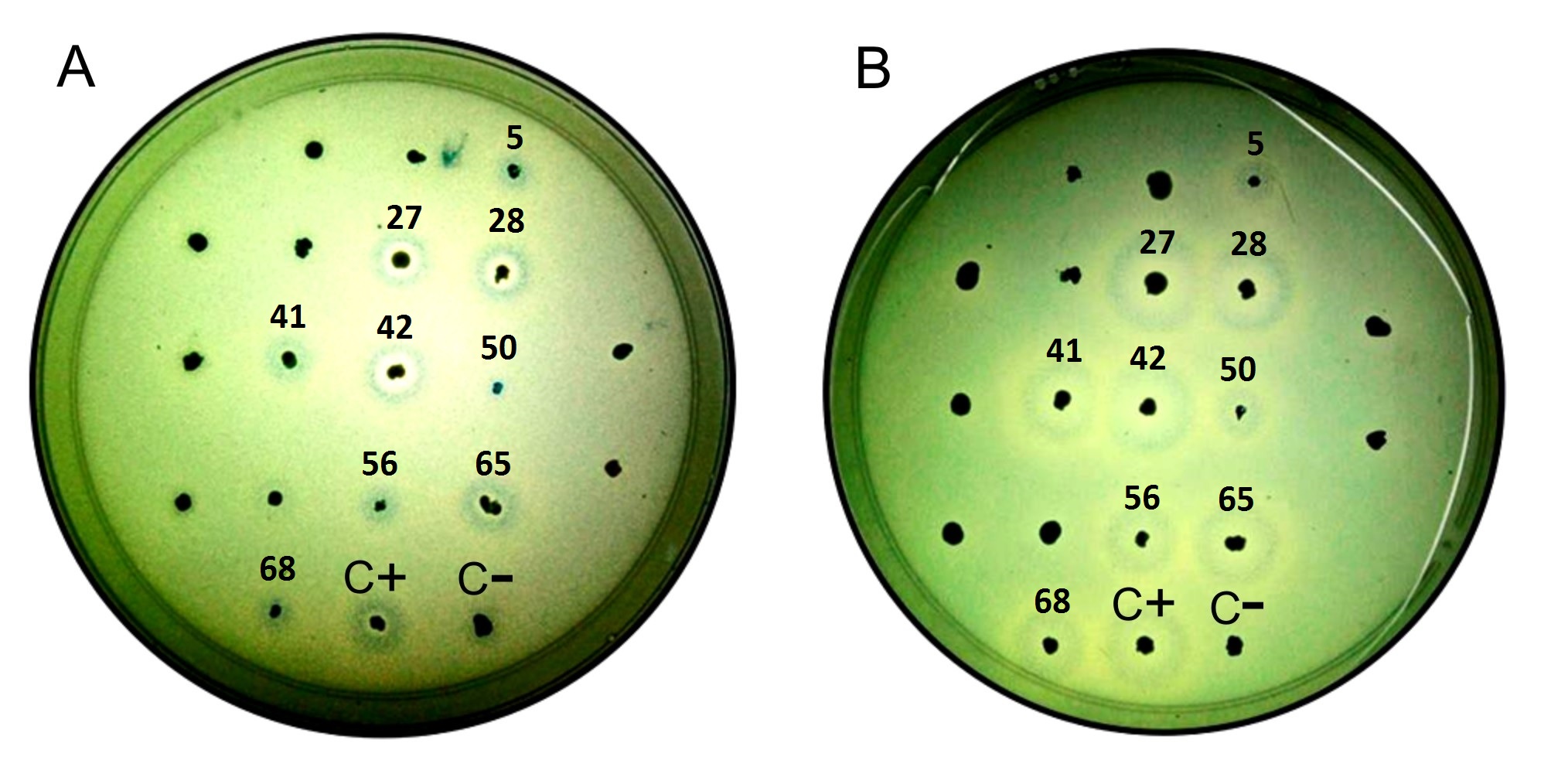

Supplement: S2 Fig — Each spot corresponds to a yeast, which is tested at the same location in both petri dishes. S. cerevisiae strain CEN.PK2-1c was used for the lawn on plates. Numbers correspond to killer strains listed on Table 1. Positive Control (C +): K. lactis AWJ137; Negative control (C-): S. cerevisiae GS1731. (JPG) [file pone.0165590.s002.jpg]

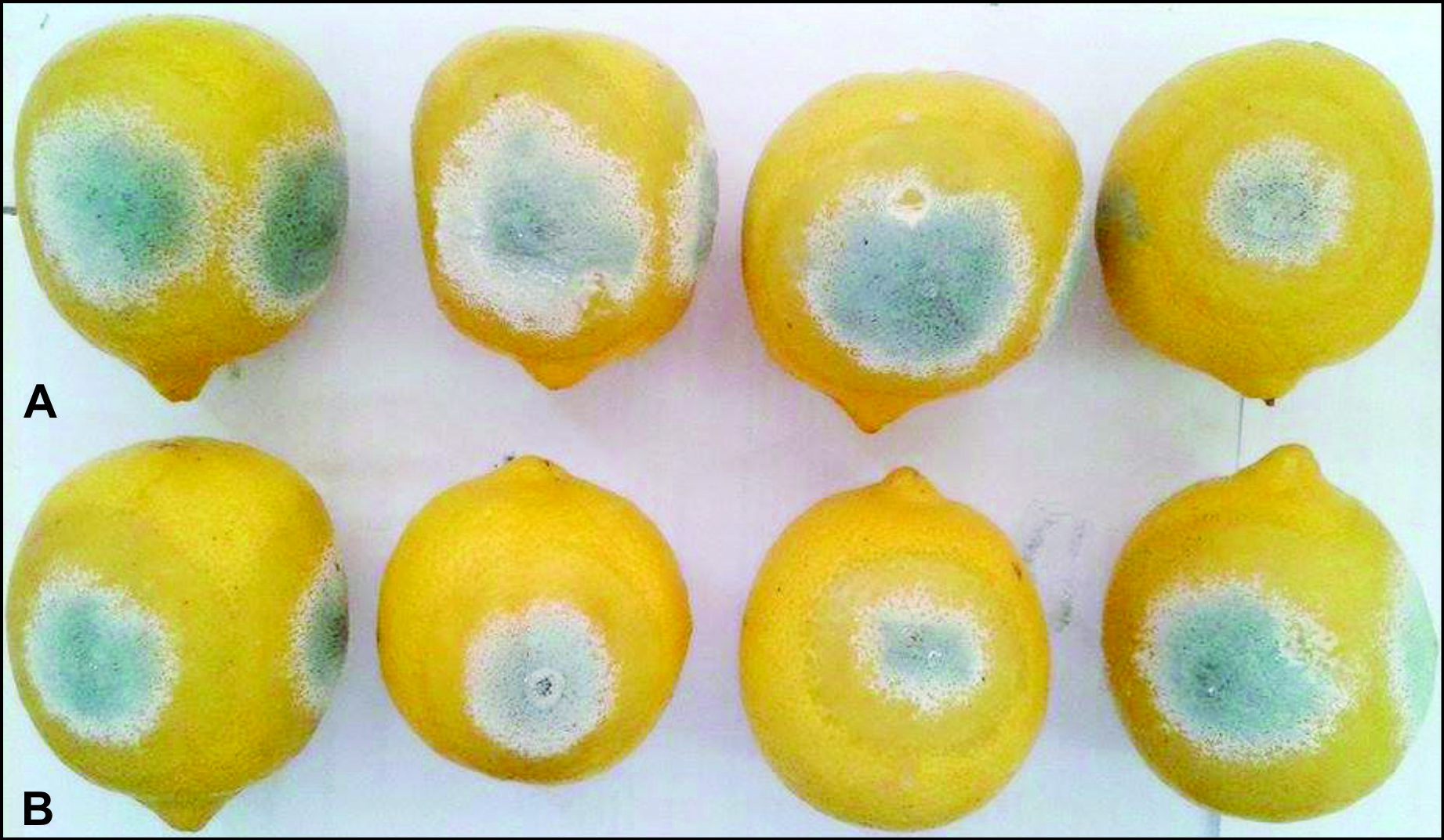

Supplement: S3 Fig — Results after 7 days of incubation. (A) Pretreated lemons with S. cerevisiae CEN.PK2-1c (no killer yeast phenotype) and then inoculated with the plant pathogen. (B) Control lemons inoculated only with P. italicum. (JPG) [file pone.0165590.s003.jpg]

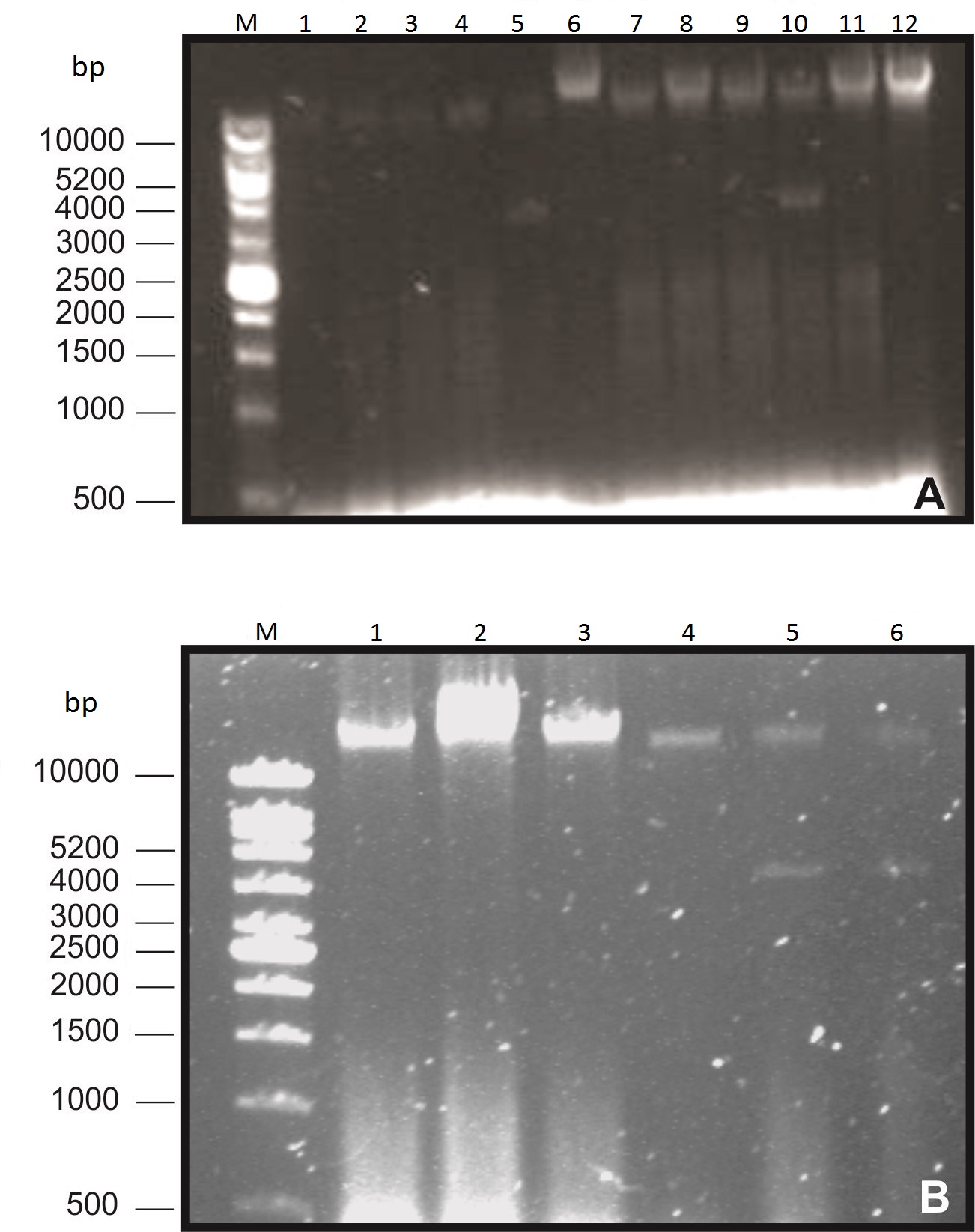

Supplement: S4 Fig — M: molecular marker (1 kb DNA Ladder, Genbiotech). (A) Lane 1, yeast 5; 2, 27; 3, 28; 4, 41; 5, 42; 6, 56; 7, 120; 8, 122; 9, 124; 10, 125; 11, 132; 12, 146. (B) Lane 1, yeast 50; 2, 73; 3, 95; 4, 123; 5, 137; 6, 145. (JPG) [file pone.0165590.s004.jpg]
