# Supplementary material for: Native Killer Yeasts as Biocontrol Agents of Postharvest Fungal Diseases in Lemons
Source: PLoS One. 2016 Oct 28;11(10):e0165590. doi: 10.1371/journal.pone.0165590 (PMC5085023; doi:10.1371/journal.pone.0165590)
Supplement: S1 Table — (DOCX) [file pone.0165590.s005.docx]

**Table S1.** Isolation source and number of strains isolated.

| **Source** | | **Number of strains** | **Total** |
| --- | --- | --- | --- |
| **Lemon** | Leaves | 47 | 87 |
|  | Fruits | 40 |  |
| **Orange** | Leaves | 42 | 83 |
|  | Fruits | 41 |  |
| **Tangerine** | Leaves | 43 | 84 |
|  | Fruits | 41 |  |
| **Grapefruit** | Leaves | 13 | 23 |
|  | Fruits | 10 |  |
| **Lemon wash water** |  | 160 | 160 |
| **Total** | | 437 | 437 |
