# Supplementary material for: Native Killer Yeasts as Biocontrol Agents of Postharvest Fungal Diseases in Lemons
Source: PLoS One. 2016 Oct 28;11(10):e0165590. doi: 10.1371/journal.pone.0165590 (PMC5085023; doi:10.1371/journal.pone.0165590)
Supplement: S2 Table — (DOCX) [file pone.0165590.s006.docx]

**Table S2.** Yeast strains with killer phenotype according to the isolation source and employed method for killer activity.

| **Source** | **Eclipse assay** | **Diffusion assay** | **Diffusion assay plus 2% NaCl** |
| --- | --- | --- | --- |
| **Lemon** | - | - | - |
| **Orange** | - | - | - |
| **Tangerine** | - | - | 7 |
| **Grape fruit** | - | - | - |
| **Wash water** | 22 | 30 | 30 |
| **Total** | 22 | 30 | 37 |
