# Supplementary material for: Native Killer Yeasts as Biocontrol Agents of Postharvest Fungal Diseases in Lemons
Source: PLoS One. 2016 Oct 28;11(10):e0165590. doi: 10.1371/journal.pone.0165590 (PMC5085023; doi:10.1371/journal.pone.0165590)
Supplement: S3 Table — (DOCX) [file pone.0165590.s007.docx]

**Table S3.** Inhibitory activity of killer yeasts against human pathogenic bacteria.

| **Strain** | **27** | **28** | **56** | **120** | **137** |
| --- | --- | --- | --- | --- | --- |
| ***Staphylococcus aureus* ATCC* 25923** | - | - | - | - | - |
| ***Enterococcus faecalis***  **ATCC 29212** | - | - | - | - | - |
| ***Klebsiella pneumoniae***  **ATCC 700603** | - | - | - | + | - |
| ***Escherichia coli***  **ATCC 25922** | - | - | - | + | - |
| ***Pseudomonas aeruginosa* ATCC 27853** | - | - | - | + | - |

+: Inhibitory activity; -: No inhibitory activity; *: American Type Culture Collection
